# Acute Photobiomodulation Does Not Influence Specific High-Intensity and Intermittent Performance in Female Futsal Players

**DOI:** 10.3390/ijerph17197253

**Published:** 2020-10-04

**Authors:** Izabela Aparecida dos Santos, Marina de Paiva Lemos, Vitória Helena Maciel Coelho, Alessandro Moura Zagatto, Moacir Marocolo, Rogério Nogueira Soares, Octávio Barbosa Neto, Gustavo R. Mota

**Affiliations:** 1 Exercise Science, Health and Human Performance Research Group, Department of Sport Sciences, Institute of Health Sciences, Federal University of Triangulo Mineiro (UFTM), Uberaba 38025-350, Brazil; izabelaeduca94@hotmail.com (I.A.d.S.); octavio.neto@uftm.edu.br (O.B.N.); grmotta@gmail.com (G.R.M.); 2 Exercise Physiology in Health and Human Performance Research Group, Department of Physical Education, University of Uberaba (UNIUBE), Uberaba 38055-500, Brazil; marina_plemos@hotmail.com; 3 Department of Physiotherapy, Federal University of Triangulo Mineiro (UFTM), Uberaba 38025-350, Brazil; vhmcoelho@gmail.com; 4 Department of Physical Education, Laboratory of Physiology and Sport Performance (LAFIDE), School of Sciences, Sao Paulo State University (UNESP), Bauru 17033-360, Brazil; azagatto@yahoo.com.br; 5 Physiology and Human Performance Research Group, Department of Physiology, Federal University of Juiz de Fora, Juiz de Fora 360360-900, Brazil; 6 Dalton Cardiovascular Research Center, University of Missouri, Columbia, MO 65211, USA; rnrmq@missouri.edu

**Keywords:** team-sports, fatigue, gender, laser, soccer, ergogenic aids

## Abstract

The acute improvement of performance after photobiomodulation therapy (PBMT) has been reported in different types of exercise. However, the effect on high-intensity and intermittent exercises that are relevant for team sports is unknown. Thus, we evaluated the effect of prior acute application of PBMT on high-intensity and intermittent exercise performance, muscle oxygenation, and physiological/perceptual indicators in amateur female futsal players. Thirteen players (24.1 ± 3.7 years) performed a testing battery (countermovement jump (CMJ), Illinois agility and YoYo intermittent recovery test level 1 (YYIR1)) preceded by 15 min of PBMT (1 min 30 s each muscular point; five muscular points in each lower limbs) or 15 min of placebo (SHAM), in a counterbalanced randomized cross-over design (one-week in-between PBMT/SHAM). All test performance did not differ (*p* > 0.05) between PBMT and SHAM, as well as blood lactate, rating of perceived exertion, heart rate, and muscle oxygenation (via near infrared spectroscopy) responses. The acute application of PBMT prior to a physical testing battery does not influence high-intensity and intermittent exercises performance, neither physiological nor perceptual responses in amateur female futsal players.

## 1. Introduction

Historically, photobiomodulation therapy (PBMT) using low level laser in clinical experiments has demonstrated relevance for wound healing (acute and chronic) by increasing collagen synthesis, and the improvement of neovascularization and angiogenesis [1]. Nowadays, PBMT has also been investigated as a possible ergogenic aid for exercise performance [2].

It has been suggested that PBMT could have positive effects on the mitochondrial enzymatic machinery for ATP production [3], leading to a potential higher phosphocreatine re-synthesis [4]. A better phosphocreatine re-synthesis could supply the necessary energy for the re-synthesis of the ATP, enhancing the next exercise bout in activities involving repeated sprints or during high-intensity exercise [5]. A study showed that PBMT significantly increased the number of repetitions (~14.5%) in a maximal voluntary contraction test of upper limbs in volleyball athletes. The authors suggested that PBMT may have increased the capacity of phosphocreatine resynthesis and consequently the force rate [6]. The potential effects of PBMT on phosphocreatine re-synthesis makes it a promising intervention for improving performance in high-intensity exercise with short pause rest and team sports, such as futsal [7].

Recently, studies have also found positive effects of acute PBMT application on endurance exercise performance [8,9]. For instance, a study showed that PBMT increased the exhaustion time in an incremental treadmill test by ~15 s (697 s placebo vs. 711 s PBMT) and the maximal oxygen uptake by ~2.3%. Additionally, the same study found a reduction in oxidative stress markers (i.e., superoxide dismutase and catalase) [8]. Another study showed the PBMT application prior to incremental treadmill test increased the distance covered by ~6.2% (or ~120 m) and the time until exhaustion by ~38 s, suggesting therefore a consistent positive effect from PBMT on endurance performance [9].

Although the mechanisms underlying the benefits of PBMT in endurance performance are not fully elucidated, it seems that mitochondria metabolism may play a crucial role [4,10]. It has been suggested that the light emitted from the PBMT device might activate the mitochondrial complex IV (COX) increasing the electron flows in the respiratory chain and consequently expanding the amount of H^+^ [10]. This would increase the availability of energy (ATP) to the cellular activities [10], thus likely collaborating to better exercise performance [11].

Even though the findings of studies assessing the effects of PBMT on performance of several exercise modalities are compelling, whether or not PBMT will improve the performance of activities involving high-intensity intermittent exercise, including changes in direction, acceleration, and deceleration, which are relevant for team sports, is still unknown [12]. Therefore, we evaluated the effects of the acute PBMT application on high-intensity and intermittent exercise performance, muscle oxygenation, physiological/perceptual indicators, and acute recovery in female futsal players. We hypothesized that acute PBMT application would improve the performance in a physical testing battery involving high-intensity intermittent exercise, changes in direction, acceleration, and deceleration.

## 2. Materials and Methods

### 2.1. Participants and Ethical Care

Thirteen female futsal players participated in this study (24.1 ± 3.7 years; 63.6 ± 8.0 kg, 1.61 ± 0.4 m, 27.9 ± 4.4% body fat; time of futsal experience 13.0 ± 4.3 years; 3-h weekly training load). The body mass and height were measured using a mechanical scale (Welmy^®^ 110 CH, Santa Bárbara d’Oeste, SP, Brazil). The body fat was obtained via bioimpedance (Biodynamics 310^®^, New York, NY, USA). The inclusion criteria were: age from 18 to 30 years, absence of chronic diseases, abstinence from the use of supplements and/or medications with potential effects on physical performance, be practicing futsal regularly for at least 1 year continuously, and do not have acute and/or recent injuries. This study was approved by the local institutional Ethics Committee for Human Experiments (Federal University of Triangulo Mineiro: 993.636) and conducted according to the Declaration of Helsinki. Written informed consent was obtained from all participants. The sample size calculation was performed a priori based on studies of PBMT in sportsmen [8,13] (effect size: 0.8; test power: 0.8), we considered the β value of 20% and α 5%, then the result of *n* = 10 was found. To counteract any potential dropouts, a sample of 13 participants was recruited for this study.

### 2.2. Experimental Design

**Figure 1:**
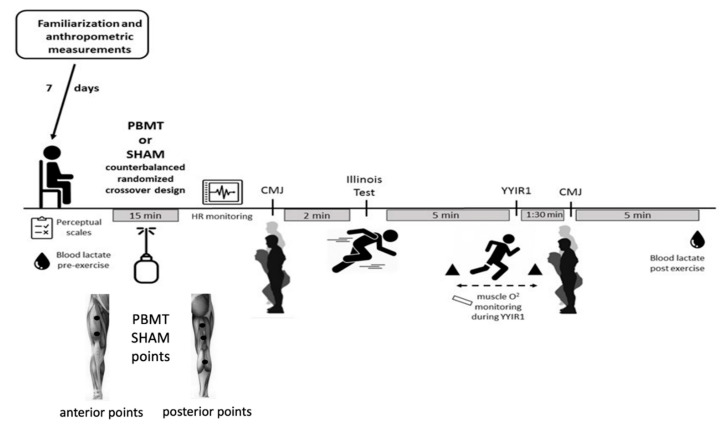
Experimental design scheme. PBMT = photobiomodulation therapy; SHAM = placebo of PBMT; perceptual scales to register perceived recovery status (PRS) and visual analogue scale (VAS) of muscle soreness; HR = heart rate, CMJ = countermovement jump; YYIR1 = YoYo intermittent recovery level 1. Illustration of the photobiomodulation therapy (PBMT) application points (bottom of the figure), two in the quadriceps, two in the hamstrings and one point in the gastrocnemius. The parameters of the PBMT were: number of diodes: 69; wavelength: mixed, that is, 34 diodes (660 nm) and 35 diodes (850 nm); laser frequency: continuous; cluster area: 44.2 cm^2^; optical output: 53 mW/cm^2^; spot site area: 0.234 cm^2^; dose: 200 J; energy density: 4.5 J/cm^2^.

Figure 1 shows the general experimental design. In a counterbalanced randomized cross-over, placebo-controlled and double-blind design, the participants performed a battery of tests preceded by PBMT or placebo (SHAM) (one week in-between). In the first experimental session, six players received the PBMT treatment, and seven received the SHAM treatment. In the second (and last) experimental session, the treatments were inverted. Two familiarization sessions (on different days) were performed for the purpose of learning and understanding of scales and tests from the study, and the anthropometric measurements were collected. Upon arriving in the laboratory, the player reported her perceived recovery status (PRS) and visual analogue scale (VAS) of muscle soreness. A blood sample (25 μL) was collected from the finger for lactate concentration measurement, then the participant rested for five minutes. Subsequently, the participant received an application of the PBMT or SHAM (PBMT device turned off).

At the end of the application of PBMT or SHAM, the player performed a battery of tests (countermovement jump (CMJ), Illinois agility test, and YoYo intermittent recovery test level 1 (YYIR1)). CMJ is commonly used as a load control parameter because it does not interfere with subsequent activities. In team sports, it is common in a single training session for athletes to train more than one goal (e.g., maximal strength plus anaerobic endurance) [14]. Based on this information, the battery of tests was developed respecting the sequence of the ‘neural’ test (CMJ) for those with ‘metabolic’ characteristics (agility and YYIR1) to minimize interference between them [15]. The details of the tests are described below. Each participant was evaluated individually to prevent possible influence from others and to be consistent.

The tests were monitored by the same experienced researcher, in the same environment (26 °C ~50% relative humidity) and time of the day. Participants were informed that both PBMT and SHAM could optimize performance (that is why here we call SHAM a placebo), and none could cause harm. The tester was blind about the condition that the players had been submitted (PBMT or SHAM), and the players had no access to the performance data (e.g., distance covered, height of the jumps) and others indicators such as blood lactate concentration or heart rate (HR) [16]. The participants were prohibited from intaking caffeinated beverages, alcohol or some other substance that could interfere in the performance for 48 h before each test session and to prevent strenuous exercises.

#### 2.2.1. Perceived Recovery Status and Muscle Soreness

To guarantee similar recovery condition, prior to both sessions, the players indicated a score on a perceived recovery scale that consists of numbering from 0 to 10 (arbitrary units (AU), where 0 corresponds to “very poorly recovered/extremely tired”, and 10 “very well recovered/with great energy” [17]. Additionally, the volunteers indicated the level of generalized muscle soreness from a VAS, which comprises a ruler from 0 to 10, in which 0 is the “absence of pain” and 10 “maximum pain” [18].

#### 2.2.2. Blood Lactate Concentration, Heart Rate and Rating of Perceived Exertion (RPE)

Blood samples (25 μL) were collected from the tip of the index finger at two moments: before PBMT or SHAM (baseline) and 5 min after YYIR1, to measure the lactate concentration using a valid portable analyzer (Accutrend^®^ Plus System, ROCHE, Basel, Switzerland). The HR was monitored during and after (3 min after the battery of tests) using the (Polar Team System^®^, Kempele, Finland) heart monitor. After the battery of tests, as well as 20 min after the session, each player indicated a score for her perceived exertion through the CR-10 Borg scale. This scale ranges from 0 to 10, where 0 is “very easy” and 10 is “very, very difficult (maximum)” [19].

#### 2.2.3. Photobiomodulation Therapy and Placebo (SHAM) Protocols

After five minutes of passive rest, PBMT/SHAM application was initiated. The application process was performed in complete cluster contact with the skin using an LED red and infrared (THOR^®^ Photomedicine, London, UK) device properly calibrated by experienced technicians. The duration was 1 min and 30 s (previously calculated to deliver the exact total energy dose = 200 J) in five points in each lower limb (two in the quadriceps, two in the hamstrings and one point in the gastrocnemius), justified by the main muscle groups used to perform the tests, resulting in 15 min in total (Figure 1; bottom) [20]. In both PBMT and SHAM, the players were “blinded” (black cloth band overlapped over the eyes) and could not hear any sound from the PBMT device.

The parameters of the PBMT were: number of diodes: 69; wavelength: mixed, that is, 34 diodes (660 nm) and 35 diodes (850 nm); laser frequency: continuous; cluster area: 44.2 cm^2^; optical output: 53 mW/cm^2^; spot site area: 0.234 cm^2^; dose: 200 J; energy density: 4.5 J/cm^2^.

The SHAM procedure (placebo) was exactly the same as the PBMT application, but with the device turned off. To blind the volunteer to the procedures, a hearing protector (sound damper) was used to avoid the sound coming from the equipment, as well as an eye protector and glasses (specific device) to avoid the visualization of light beams coming from the equipment (black cloth band overlapped over the eyes also).

#### 2.2.4. Battery of Physical Tests

##### Countermovement Jump Test

The vertical jump was performed in two moments: 30 s after the application of PBMT or SHAM and 1 min after the YYIR1, to evaluate if the PBMT would be able to reduce acute fatigue. Three attempts (with 10 s of interval in-between) were performed. The highest jump height was considered for analysis. The player performed the CMJ on a force-platform (Bertec Acquire^®^, Columbus, OH, USA). Players were asked to keep their hands on their hips to prevent influence of arm movements on the vertical jump and to squat down to approximately 90° by flexing at the knee and hip and then immediately extend the knees and hip to jump high. After the voice command, the jump was executed. Before, we communicated that the jump should be as high as possible and the feet should touch the platform with the knees extended [21]. The software used to interpret CMJ data was the OriginPro 8 version 2018 for Windows^®^ (Northampton, MA, USA).

##### Illinois Agility Run Test

The Illinois agility test was performed 30 s after the CMJ, in an area with four cones (10 m long by 5 m wide and 3.3 m between the 4 central cones) [22]. Participants were instructed to complete the course in the frontal displacement and with the highest possible speed. Three attempts with 1 min and 30 s of recovery between the trials were allowed. The total, mean, and best time to complete the three attempts were considered for analysis and recorded by a photocell system.

##### Muscle Oxygenation Measurements

During the five minutes interval between the agility test and the beginning of the YYIR1, the near-infrared spectroscopy (NIRS) device was setup. Based on the aerobic energy contribution [23] when performing the YYIR1 test (~190 s in the familiarization sessions) and potential effects of PBMT on mitochondrial pathways [10], we assessed the local oxygenation of the quadriceps vastus lateralis (VL) by NIRS as previously described [24]. Briefly, a wireless near-infrared spectroscopy (NIRS) system (Moxy Muscle OxygenSensor; Moxy Monitor^®^, Hutchinson, MN, USA) probe was place on the vastus lateralis of the right muscle. This system functions by sequentially sending light waves (630–850 nm) from 4 light-emitting diodes into the muscle of interest and recording the amount of received light at 2 detectors. The detectors are positioned at a distance of 12.5 and 25 mm from the light source, allowing the system to calculate muscle oxygenation with a penetration depth of about 12 mm. Muscle oxygen saturation was estimated as follows: first, a 30 s average of resting muscle oxygen saturation baseline prior to the test was calculated. Then, the area above the curve of the oxygen saturation signal during the exercise was calculated [25]. The total amplitude of blood volume (∆tHb_Ex_) was calculated by the difference between the maximum and minimum muscle oxygen saturation (SmO2) reached during exercise [24]. It is worthwhile to highlight that the two PBMT application points in the quadriceps muscle (Figure 1; bottom) are enough to spread the light to the spot measured by the NIRS device [26].

##### YoYo Intermittent Recovery Level 1 (YYIR1)

YYIR1 measures the ability to perform an intense and intermittent exercise, such as in futsal. The YYIR1 is highly reproducible, low cost, effective and reliable, including for females, and provides useful data [27]. After five minutes of the agility test, the YYIR1 was started. The test consists of repeated shuttle runs (2 × 20 m) and progressively increasing velocity stages (initial velocity 8 km·h^−1^) that are guided by specific audio (10 s for recovery in a marked area 5 m behind the finish line). Test closure occurred due to lack of reach in the footage and/or rhythm twice. The audio was in a language different from that spoken by the players (keeping them blind about the performance) [28]. Verbal stimuli during the execution were standardized and performed by the tester.

### 2.3. Statistical Analysis

The Shapiro–Wilk test was applied to verify the distribution of the data. For between protocol analysis (SHAM vs. PBMT) we used the paired *T* test (parametric data). For CMJ (pre-and post-test) we applied a one-way ANOVA (time and treatment). The significance level was set at 0.05. To evaluate an individual effect from PBMT on the YYIR1, we calculated the smallest worthwhile change (SWC). We calculated the SWC by multiplying the standard deviation of the distance covered in the YYIR1 in the SHAM by 0.6.

## 3. Results

The perceived recovery scores did not differ (*p* = 0.99) between SHAM (8.46 ± 0.89 AU) and PBMT (8.48 ± 0.96). The muscle soreness scores also did not differ (*p* = 0.33) between SHAM (1.23 ± 0.84 AU) and PBMT (0.84 ± 0.68).

The height of CMJ after receiving treatment (SHAM and PBMT), as well as after the battery of tests did not differ: pre = 18.5 ± 1.7 cm, post = 18.9 ± 19 cm (*p* = 0.17) vs. pre = 19.3 ± 2.8 cm, post = 19.3 ± 3.0 cm (ANOVA, interaction and main effects: F_3,48_ = 0.30893; *p* = 0.818), respectively.

The agility test results did not differ (*p* > 0.05) between PBMT vs. SHAM, total time (s) PBMT (58.9 ± 2.3) vs. SHAM (58.7 ± 3.1) *p* = 0.99, mean time (s) PBMT (19.6 ± 1.0) vs. SHAM (19.6 ± 0.7) corresponding to *p* = 0.99, finally best time (s) PBMT (19.3 ± 1.0) vs. SHAM (19.5 ± 0.9) *p* = 0.68. 

**Figure 2:**
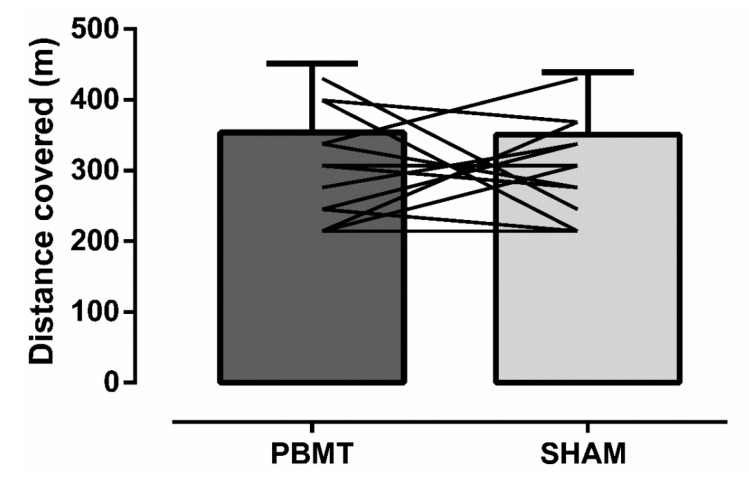
Distance covered during the YYIR1 test did not differ (*p* = 0.93; paired *T* test) between photobiomodulation therapy (PBMT) vs. placebo (SHAM). Data are presented as mean ± SD; *n* = 13. Lines represent individual data. According to the smallest worthwhile change (SWC) calculation (i.e., SHAM SD × 0.6 = ~53 m), 1 player performed better after PBMT; 1 player performed better after SHAM and 11 players had the same performance between PBMT vs. SHAM.

Figure 2 shows that the distance covered in the YYIR1 did not differ (*p* = 0.93) between PBMT (353.8 ± 97.8 m) vs. SHAM (350.8 ± 88.2 m). The time to perform the YYIR1 also did not differ (*p* = 0.96) between PBMT (195 ± 77 s) and SHAM (191 ± 46 s). The SWC calculated for the YYIR1 was ~53 m in the YYIR1 (SHAM SD = 88.2 m × 0.6 = 52.9 m or ~53 m). If the individual distance covered was higher than 53 m, it was classified as an improvement. If the individual distance covered in the YYIR1 was lower than 53 m, it was classified as a decrement on YYIR1 performance. Figure 2 shows that after PBMT, one out of the 13 players covered higher distance (~7.7%), while one covered lower distance (~7.7%) after PBMT, and 11 maintained the distance covered in YYIR1 (~84.6%).

Blood lactate concentration did not differ (*p* > 0.05) between PBMT and SHAM either at baseline as well as post-YYIR1 (see specific *p* values in Table 1). The heart rate responses and RPE to the YYIR1 test are presented in Table 1, showing no difference between SHAM and PBMT.

**Figure 3:**
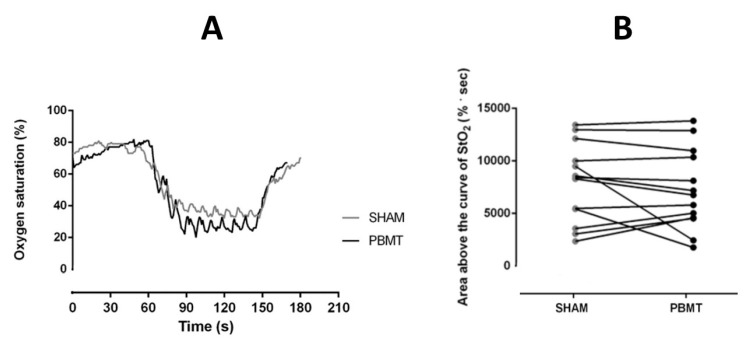
(**A**) Oxygen saturation during the YYIR1 test did not differ (*p* > 0.05) between PBMT and placebo (SHAM). Data are presented as mean; *n* = 13. (**B**) Area above the curve of tissue oxygen saturation (StO2) of individuals.

No difference was found between conditions in ∆tHb_Ex_ (*p* = 0.27) PBMT (58.3 ± 15.8 µM) vs. SHAM (52.9 ± 9.1 µM). Figure 3A shows oxygen saturation during YYIR1 in both conditions (PBMT and SHAM) without significant differences (*p* > 0.05). Figure 3B shows the area above the tissue oxygen saturation (StO2) curve of the 13 individuals.

## 4. Discussion

The purpose of this study was to evaluate the acute effect of PBMT on a physical testing battery involving high-intensity, intermittent and neuromuscular exercise performance in female futsal players. Our main finding was that acute PBMT did not influence any performance (agility or YYIR1) or recovery (CMJ) parameter, physiological (HR, blood lactate concentration, and muscular oxygenation) or perceptual indicators. This study is the first to test the effects of PBMT on these types of exercises, which may be relevant for team sports.

Our results show that the perceived recovery and muscle soreness scores were similar for the two experimental sessions, ensuring primary conditions for comparison between SHAM vs. PBMT.

CMJ has been used as a practical and useful tool for monitoring neuromuscular fatigue and also to evaluate physical recovery [29]. This result is in line with another study, where no improvement in CMJ performance was found with the application of PBMT after intense exercise performed on an ergometric cycle [20]. The higher soccer-induced muscle damage and fatigue from a real match are probably higher than the disturbance caused by the battery of tests applied in our study [27], which explains the non-reduction in CMJ performance before and after exercise. However, the use of specific tests instead of the game itself is more advantageous in relation to the control of variables (i.e., running speed), which in the game is unpredictable.

The enhancement of mitochondrial activity is one of the most discussed mechanisms for PBMT, which may lead to a higher resynthesis of phosphocreatine [4]. To restore 100% of the phosphocreatine used in a previous bout of high-intensity exercise at least 3 to 5 min is necessary [30]. Therefore, we tested if PBMT could improve the agility test with an insufficient pause rest (i.e., 1 min 30 s). Our data showed that PBMT had no effect on the Illinois agility test as well as in the HR and RPE responses. We did not find any similar study testing PBMT in agility tests specifically. Future studies should test a higher number of repetitions to check for a potential improvement in longer bouts of high-intensity exercise with short pause rest durations.

Futsal matches demand acceleration, deceleration, changes in direction several times, resembling the patter of the YYIR1 [31]. As YYIR1 has a relevant aerobic component and PBMT has been a promising tool to enhance aerobic metabolism [8,9], we sought to check if PBMT could affect the performance of the YYIRT1. Our data showed that PBMT did not influence the performance and physiological/perceptual indicators of the YYIR1 (i.e., HR, lactate concentration, and muscle oxygenation responses and RPE). Even considering the individual responses through SWC, which may be relevant in studies evaluating ergogenic aids [32] (i.e., not only mean group), our results show no differences between PBMT and placebo (Figure 2). This is the first study testing the effects of PBMT on the YYIR1 performance in female futsal players. The distance found here (~350 m) is considerably lower than female professional soccer players (~972 m) [33], probably due to the training status, and the higher distances covered during the soccer (vs. futsal) matches and training sessions, which demand higher levels of aerobic metabolism [33].

It has been suggested that the light emitted from PBMT would act in the mitochondria [3,10,11,34]. The light activation of the mitochondrial COX (complex IV) would increase the electron flows in the respiratory chain, expanding the amount of H^+^ and, consequently, the ATP availability to the muscle cells [10,11]. In the current study, we sought to evaluate if such alterations might improve endurance performance (i.e., YYIR) or benefit muscle oxygen saturation. Our results show that responses of muscle oxygenation during YYIR1 (measured through the NIRS) did not show any difference between PBTM vs. SHAM (Figure 3). Between the first minute (60 s) and the third minute (180 s) of the YYIR1, there was a drastic decrement in the oxygen saturation (Figure 3). Although there may be an influence of the flow on the saturation signal, no differences were found in the response of total hemoglobin during YYIR1 between PBMT vs. SHAM, thus, the similarity of the saturation drop in the conditions was due to the non-difference in oxygen extraction. The decrement on the oxygen saturation during the YYIR1 was already expected, because during incremental physical tests (such as the YYIR1), the rate of increase in muscle metabolic demand, and consequently oxygen consumption, may overcome changes in local oxygen delivery, thereby reducing muscle oxygen saturation levels [35]. We highlight that the two PBMT application points in the quadriceps muscle (Figure 1; bottom) in the current study are enough to spread the light to the spot measured by the NIRS device [26].

The values of RPE, blood lactate concentration and HR of the current study proved that the effort performed during the YYIR1 was near maximum (e.g., RPE = ~9.5 AU, lactate > 8 mmol·L^−1^ and HRmean = ~87%) in both PBMT and SHAM conditions (Table 1), supporting the validity of our experimental design. The RPE of the session (i.e., all battery of tests) did not differ between PBMT and SHAM (~7.5 AU), which means that the players felt the session was “very hard/intense”. Unlike our data, another study showed that the PBMT reduced the RPE during submaximal running exercise and improved some performance and physiological variables (e.g., running economy and total time to exhaustion). The discrepancy between our RPE findings and their RPE findings may be due to the kind of exercise test performed (e.g., here, intermittent involving changes of direction and maximum vs. running straight, submaximal) [2]. Although the menstrual cycle phase does not affect the performance of the YYIR1 [36], we had no female player reporting being in the menstrual period during the study. There is no study that has effectively analyzed the effect of PBMT on HR responses during an intermittent exercise test. However, there is speculation that the application of PBMT may induce cardiovascular efficiency by increasing the muscle oxygen extraction at submaximal intensities, decreasing or maintaining HR values [2]. In the present study, we found no effect from PBMT on HR responses during the YYIR1.

Blood lactate concentration above 8 mmol·L^−1^ is one of the indicators of maximum effort [37]. In the current study, after the YYIR1 the blood lactate values were ~11.1 to ~13.5 mmol·L^−1^, which means the tests were maximum (for our sample). The effects of PBMT on post-exercise lactate concentration are still quite controversial [38,39]. A possible benefit would be due to the increase in mitochondrial activity, resulting in an increase in lactate concentration removal [4]. Studies show that the application of PBMT before high intensity exercises increases lactate removal [7,40]. However, other studies corroborate our findings, showing no effect from PBMT on blood lactate concentration [38,39].

Through our methods, it is not possible to explain the mechanisms for the present divergences in the literature in relation to the use of PBMT; however, studies that also did not find performance optimization and some change in physiological variables suggest that these contradictions may be linked to the studied population (i.e., age, sex, training level), different types of exercises (i.e., jumping, resistance runs, weight exercises), and mainly the difference in the parameters of use of PBMT [20,41,42].

In this study, PBMT was applied to five muscular points. This distribution was used starting from the light spreading capacity in the tissue [26]; therefore, the chosen points were able to stimulate the main muscle groups used during the exercises. The main issue and controversies in studies involving PBMT and exercise performance have been the “ideal dose”. Although we have used a dose of 4.5 J/cm² (200 J of energy) accordingly a meta-analysis [43], we acknowledge that the “ideal dose” is still unknown. Additionally, the time window to present an effect may vary, as it occurs with other ergogenic aids [44] and PBMT [11]. Therefore, we do not know the effects from other doses on the exercises similar to those investigated here.

## 5. Limitations

Although we informed the players about all procedures during the whole experiment, and we measured the perception of the recovery and muscle soreness before each testing session, we acknowledge that it is challenging to ensure a well-controlled recovery (e.g., quantity and quality of sleep between the two experimental sessions). Another potential limitation is the accumulation of fatigue from one exercise test to the next, even though the battery was designed and performed (e.g., rest time in-between) to minimize these possible effects.

## 6. Conclusions

The acute application of PBMT prior to exercises does not influence performance in specific high-intensity and intermittent exercise tests, neither physiological responses (i.e., HR, muscle oxygenation, and blood lactate concentration) or perceptual intensity (i.e., RPE) in amateur female futsal players.

## Figures and Tables

**Table 1:** Blood lactate concentration, heart rate (HR) responses and rating of perceived exertion (RPE) for the YYIR1 test.

	PBMT	SHAM	*p* Value
Baseline lactate (mmol·L^−1^)	1.8 ± 0.8	2.1 ± 0.6	0.30
Post-YYIR1 lactate (mmol·L^−1^)	11.1 ± 2.9	13.5 ± 3.8	0.10
HR min (bpm)	131.4 ± 24.1	132.2 ± 17.0	0.92
HR mean (bpm)	171.1 ± 16.0	169.4 ± 12.5	0.76
HR peak (bpm)	190.3 ± 8.1	184.8 ± 10.0	0.10
%HRmax	87.2%	86.2%	--
RPE (AU)	9.46 ± 0.7	9.38 ± 0.6	0.79
HR rec 1 min	119.2 ± 14.4	112.8 ± 13.1	0.30
HR rec 2 min	117.1 ± 12.1	111.6 ± 11.2	0.28
HR rec 3 min	112.6 ± 13.1	110.6 ± 11.1	0.69

Data are mean ± standard deviation; *n* = 13; arbitrary units (AU); photobiomodulation therapy (PBMT); SHAM (placebo). %HRmax = percentage values of maximum heart rate (220-age) over the HR mean.
